# A call for increased inclusivity and global representation in pharmacogenetic testing

**DOI:** 10.1038/s41525-024-00403-1

**Published:** 2024-02-22

**Authors:** April Kennedy, Gabriel Ma, Roozbeh Manshaei, Rebekah K. Jobling, Raymond H. Kim, Tamorah Lewis, Iris Cohn

**Affiliations:** 1 Division of Clinical Pharmacology and Toxicology, Department of Paediatrics, The Hospital for Sick Children, University of Toronto, Toronto, ON Canada; 2 Program in Translational Medicine, The Hospital for Sick Children, Toronto, ON Canada; 3 University of Toronto, Toronto, ON Canada; 4 Cardiac Genome Clinic, Ted Rogers Centre for Heart Research, The Hospital for Sick Children, Toronto, ON Canada; 5 Division of Clinical and Metabolic Genetics, The Hospital for Sick Children, Toronto, ON Canada; 6 Genome Diagnostics, Department of Paediatric Laboratory Medicine, The Hospital for Sick Children, Toronto, ON Canada; 7 Fred A. Litwin Family Centre in Genetic Medicine, University Health Network, Department of Medicine, University of Toronto, Toronto, ON Canada; 8 Division of Neonatology, Department of Paediatrics, The Hospital for Sick Children, Toronto, ON Canada

**Keywords:** Pharmacogenetics, Genetics

## Abstract

Commercial pharmacogenetic testing panels capture a fraction of the genetic variation underlying medication metabolism and predisposition to adverse reactions. In this study we compared variation in six pharmacogenes detected by whole genome sequencing (WGS) to a targeted commercial panel in a cohort of 308 individuals with family history of pediatric heart disease. In 1% of the cohort, WGS identified rare variants that altered the interpretation of metabolizer status and would thus prevent potential errors in gene-based dosing.

Pharmacogenetic (PGx) testing can be employed to optimize drug selection and dosing by factoring in genetic influences on drug metabolism and response, reducing side effects and enhancing therapeutic outcomes compared to a generic “one-size-fits-all” treatment approach^1,2^. Pharmacogenes, such as the family of cytochrome P450 (CYP) enzyme-encoding genes are highly polymorphic, with significant variation observed across various racial and ethnic groups^3–5^. Rare variants account for roughly 30–40% of functional PGx variation^6^. Ensuring that PGx testing captures these variations within the global population is essential for providing precise and reliable gene-drug-based dosing recommendations^5,7^.

Targeted genotyping and sequencing have catalyzed PGx testing due to the availability of low-cost commercial panels relative to whole genome sequencing (WGS)^8,9^. While uptake of commercial PGx panels has expanded access to testing, targeted genotyping assays in particular, capture just a fraction of the variation that influences response and adverse reaction to medications^5,8^. This pitfall is pronounced when targeted PGx genotyping panels are applied to individuals of non-European descent^5^. The disparity in representation can be attributed to factors such as the reference genome and historic disproportionality of European ancestry in genetic research^3,10^. Despite the widespread use of human reference genome GRCh38, ~70% of GRCh38.p13 is derived from a single individual, which results in an inadequate representation of genetic diversity^10^. Consequently, this limitation hinders a comprehensive understanding of population-specific variation and the accurate sequencing of polymorphic regions^10,11^. Another significant concern arises from the overrepresentation of European ancestry in PGx research, in contrast to the under-documentation of clinically actionable alleles that are relatively common in other genetic ancestral groups^3^. Nevertheless, functionally characterized, clinically actionable variants with low general population frequency are also often omitted from panels^12^. Collectively, the gap in representation reflected in commercial PGx panels may further exacerbate health disparities in non-European origin populations^3,11^. Bridging the diversity gap will improve pharmacotherapy guidance for all patients^5,11^.

Recent studies have demonstrated the benefit of a WGS-guided approach to capture clinically actionable PGx variants in diverse populations in addition to uncovering variants that warrant functional characterization^12,13^. To further explore the utility of WGS in pharmacogenetic applications, we evaluated the concordance of PGx diplotype calls between a commercial targeted genotyping panel and WGS in a cohort of individuals with cardiac disease (*n* = 308) from diverse racial and ethnic backgrounds.

Self-reported maternal and paternal ancestry data was available for 245 (80%) study participants (Table 1). Of these participants, 84% were classified as having a single ancestry, and 16% were classified as having multiple ancestries. Among participants with a single reported ancestry, European was most frequently reported (74%), followed by South Asian (12%).

**Table 1:** Breakdown of self-reported ancestries observed within the cohort

	Ancestral background	Number of individuals
Individuals with single self-reported ancestry	African or Caribbean	13
	Central American	2
	East Asian	3
	European	152
	Indigenous or Native American	1
	Middle Eastern	6
	South American	3
	South Asian	25
	Southeast Asian	1
Individuals with multiple (>1) self-reported ancestries	African/East Asian	1
	African/European	6
	African/European/Indigenous or Native American	1
	African/European/South American	1
	African/Middle Eastern	1
	African/Southeast Asian/European/Indigenous or Native American	1
	Caribbean/European	5
	Caribbean/European/South American	1
	Caribbean/South Asian	1
	Central American/European	1
	East Asian/European	1
	East Asian/South American	1
	East Asian/Southeast Asian	1
	European/Indigenous or Native American	11
	European/Middle Eastern	1
	European/South American	2
	European/Southeast Asian	2
	South American/Southeast Asian	1

Analysis of WGS data and targeted panel calls revealed discordant haplotype assignments in three unrelated participants (denoted A–C) of different self-reported ancestral backgrounds. These individuals exhibited variants in *CYP2C9* and *CYP2C19* that were captured by WGS, but not by the commercial panel. Identification of the rare alleles in these participants by WGS consequently altered the interpretation of metabolizer status. A deletion of ~70 kb, encompassing the first four exons of *CYP2C19*, was identified in participant A of North European and Jewish Ashkenazi ancestry (Table 2). The commercial panel is designed to detect single nucleotide variants and small insertions/deletions, which explains the inability to detect the deletion in this participant. Deletions involving *CYP2C19*, such as CYP2C19*37 (partial deletion) are rare, with an estimated heterozygote frequency of 0.046% in the general population^14^. In participant B of South Asian ancestry, WGS identified CYP2C9*14, a decreased function allele (Table 2). Notably, CYP2C9*14 while globally rare, is more common among South Asian populations in gnomAD (Table 3). The commercial panel does not interrogate this allele, explaining the incorrect assignment of *1. The CYP2C9*3 allele was detected by both technologies (Table 2). Furthermore, WGS captured rare variants defining CYP2C19*34 in participant B; an allele of uncertain function without actionable clinical recommendations (Tables 2 and 3). Uncertain function alleles are not interrogated by the commercial panel and were thus assigned *1. In participant C of Jamaican ancestry, WGS identified variants defining CYP2C19*22, a rare no-function allele not interrogated by the commercial panel (Tables 2 and 3).

**Table 2:** Participant diplotype results exhibiting discordance between the commercial targeted genotyping panel and WGS

	Self-reported ethnicity	Commercial panel diplotype	Metabolizer status from the commercial panel	WGS diplotype	Metabolizer status from WGS
Participant A	North European and Jewish Ashkenazi	CYP2C19*1/*1	Normal metabolizer	CYP2C19*1/*37 [no function]^1^	Intermediate metabolizer
Participant B	South Asian	CYP2C9*3/*1	Intermediate metabolizer	CYP2C9*3/*14 [decreased function]^1^	Poor metabolizer
		CYP2C19*1/*1	Normal metabolizer	CYP2C19*1/*34 [uncertain function]^a^	Unknown
Participant C	Jamaican	CYP2C19*1/*1	Normal metabolizer	CYP2C19*1/*22 [no function]^a^	Intermediate Metabolizer

*WGS* whole genome sequencing.

^a^Functionality information in square brackets refers to the discordant allele. CYP2C9*1 and CYP2C19*1 are wild-type alleles that exhibit normal function. CYP2C9*3 is a decreased function allele.

**Table 3:** Frequencies of the star alleles captured by WGS as annotated by the Genome Aggregation Database (gnomAD) (v3.1.2)

Star allele	Defining variant(s)	African/African American (%)	East Asian (%)	European (Finnish) (%)	European (non-Finnish) (%)	Latino/admixed American (%)	South Asian (%)
CYP2C9*14	NM_000771.4:c.374G>A	0.00965	0.0386	0	0.01323	0.06556	2.094
CYP2C19*22^a^	NM_000769.4:c.557G>C	0.002414	0	0	0.01029	0	0
CYP2C19*22^a^	NM_000769.4:c.991A>T	98.75	96.23	94.94	93.66	95.37	88.56
CYP2C19*34^a^	NM_000769.4:c.7C>T	0.002413	0	0	0	0	1.121
CYP2C19*34^a^	NM_000769.4:c.10T>C	0.002412	0	0	0.004411	0	1.12

*WGS* whole genome sequencing.

^a^CYP2C19*22 and CYP2C19*34 haplotypes are defined by two variants and are thus displayed in two rows.

One ongoing challenge in the interpretation of genomic data is the paucity of knowledge about variants in various ancestral populations^3,11^. Here we demonstrate that the identification of rare alleles by WGS prevents potential errors in gene-based dosing. For example, CYP2C19*37, CYP2C19*22, and CYP2C9*14 alleles reduce the activity of their respective enzymes, thereby leading to distinct medication metabolism patterns for participants A–C. Consequently, the alleles identified by WGS altered the determination of metabolizer statuses. Reduced CYP2C19 enzyme activity in participants A and C would warrant deviation from standard dosing, or avoidance of certain medications such as the antiplatelet medication, clopidogrel^15^. Significantly reduced CYP2C9 enzyme activity in participant B would also warrant deviation from standard dosing of certain medications or selection of alternative therapies. For example, this may influence medications such as non-steroidal anti-inflammatory drugs (NSAIDs) and HMG-CoA reductase inhibitors (statins) that are broken down by this enzyme pathway^16,17^. Although dosing guidance is not available for uncertain function alleles, this finding in participant B emphasizes the critical need to functionally characterize rare variants in various racial and ethnic groups^3^.

Our findings highlight how the absence of ancestrally specific variants from a commercial panel may lead to inaccurate information for medication guidance. The discordant haplotype assignments were attributed to variant omission in the panel design and may be a result of outdated evidence. The PGx Working Group of the Association for Molecular Pathology (AMP) Clinical Practice Committee recommends a set of variants that should serve as the basis for clinical PGx panels. As per the last review of *CYP2C9* published in 2018, CYP2C9*14 was considered an allele of uncertain function^18^. A more recent review by the Clinical Pharmacogenetics Implementation Consortium (CPIC) in 2019 found moderate evidence for decreased enzyme activity associated with this allele, which is denoted by the Pharmacogene Variation Consortium (PharmVar) and the Pharmacogenomics Knowledgebase (PharmGKB). This warrants the inclusion of CYP2C9*14 in PGx panels, to expand coverage of clinically actionable PGx variants, particularly in individuals of South Asian ancestry who carry this variant more frequently. The CPIC annotated, non-functioning CYP2C19*22 allele, has been observed in individuals of African/African American ancestry in gnomAD, although it was not included in the most recent recommendations by AMP, likely attributable to low population frequency (Table 3). Deletions of *CYP2C19* are also not included in the most recent recommendations by AMP^19^. This may be a result of the scarcity of literature detailing the functional consequence of *CYP2C19* deletions. Despite this, partial and full *CYP2C19* deletions have been annotated by CPIC as ‘no function’ alleles based on the presumption of a resulting non-functional protein. Pipelines for the detection of copy number variation in pharmacogenes like *CYP2D6* have already been validated^12^. Copy number variation in *CYP2C19* is not typically assayed by targeted PGx tests, although it may be advisable for commercial and clinical laboratories to consider this based on accumulating reports of this variation in the general population^14^. Next-generation sequencing, particularly WGS, can identify variation across diverse populations and offers the ability to re-interrogate data as additional functional variants are elucidated and as new gene-drug guidance becomes available^3,12^. Until WGS is adopted in routine practice, targeted panels need to be continuously updated to reflect the rapidly evolving literature and must extend coverage to variation that is representative of diverse and under-served populations.

The significance of representative genetic testing cannot be overemphasized. The development of a comprehensive human pangenome reference serves as a crucial stride towards promoting inclusivity in genetics^10^. Likewise, commercial genetic testing panels should prioritize the detection of clinically significant PGx variants across diverse racial and ethnic groups, specifically when this information is used for clinical care. To prevent further mis-assignments of metabolizer status as observed in the individuals reported herein, ongoing evaluation of ancestrally specific genetic variants should be foundational in designing and updating genetic testing panels.

## Methods

Families with pediatric heart disease were recruited through the Ted Rogers Cardiac Genome Clinic (CGC) at a single site, The Hospital for Sick Children, Division of Cardiology. The study was conducted in collaboration with the Division of Clinical Pharmacology and Toxicology at The Hospital for Sick Children. This study was conducted in accordance with all relevant ethical regulations including the Declaration of Helsinki. The study was approved by the research ethics board of The Hospital for Sick Children (REB #1000053844 and #1000053445) and the University Health Network (REB#: 16-6282). Written informed consent was obtained on behalf of all participants. Details of the CGC cohort and ongoing PGx pilot study have been previously described^20,21^. Self-reported maternal and paternal ancestry was captured during the initial genetics assessment and pedigree analysis. Maternal and paternal ancestries were transformed into ancestral categories by investigators. Since the last publication in 2021^21^, an additional 308 participants have been enrolled as of June 2023. The results reported herein are from newly enrolled participants.

Banked DNA from whole blood was used for genetic analyses. PGx data was evaluated by WGS via the HiSeq X system (Illumina, Inc., San Diego, CA, USA) and by orthogonal targeted genotyping via the VeriDose® Core MassARRAY panel (Agena Bioscience, San Diego, CA, USA). Variants from six pharmacogenes (*CYP2D6, CYP2C9*, *CYP2C19, CYP3A5, SLCO1B1, VKORC1*) were analyzed. Evaluation of the six pharmacogenes was restricted to variation that has been annotated by PharmVar (https://www.pharmvar.org/) and PharmGKB (https://www.pharmgkb.org/). PGx haplotypes for the *CYP* genes are designated using the star (*) allele nomenclature system. In accordance with standard practice, a default assignment of *∗1* wild-type allele, or *3 for *CYP3A5*, was made if none of the tested variants were detected by the commercial panel or in the absence of WGS-identified PGx variants with known functional status^18,22^. Impact on metabolism, or metabolizer status, for each participant, was determined using established genotype-to-phenotype translation tables from CPIC. PGx haplotypes and inferred metabolizer statuses derived from WGS data were compared to those generated by the MassARRAY Typer Analyzer (version 5.0.2) and iPLEX ADME PGx Pro (version 3.99.105) software to assess concordance between the two technologies.

### Reporting summary

Further information on research design is available in the Nature Research Reporting Summary linked to this article.

### Supplementary information

Reporting Summary

## Data Availability

Additional de-identified data may be shared upon reasonable request and with fulfilled legal requirements (approval from all ethics committees and data transfer agreements).
